# Digital surveillance in Latin American diseases outbreaks: information extraction from a novel Spanish corpus

**DOI:** 10.1186/s12859-022-05094-y

**Published:** 2022-12-23

**Authors:** Antonella Dellanzo, Viviana Cotik, Daniel Yunior Lozano Barriga, Jonathan Jimmy Mollapaza Apaza, Daniel Palomino, Fernando Schiaffino, Alexander Yanque Aliaga, José Ochoa-Luna

**Affiliations:** 1 Department of Computer Science, Universidad de Buenos Aires, Buenos Aires, Argentina; 2 Computer Science Research Institute, CONICET-UBA, Buenos Aires, Argentina; 3 Department of Computer Science, Universidad Católica San Pablo, Arequipa, Peru; 4 Institute of Linguistics, Universidad de Buenos Aires, Buenos Aires, Argentina

**Keywords:** Event-based surveillance, Diseases outbreaks, Spanish corpus, Named entity recognition, Relation extraction, ProMED-mail, Digital surveillance

## Abstract

**Background:**

In order to detect threats to public health and to be well-prepared for endemic and pandemic illness outbreaks, countries usually rely on event-based surveillance (EBS) and indicator-based surveillance systems. Event-based surveillance systems are key components of early warning systems and focus on fast capturing of data to detect threat signals through channels other than traditional surveillance. In this study, we develop Natural Language Processing tools that can be used within EBS systems. In particular, we focus on information extraction techniques that enable digital surveillance to monitor Internet data and social media.

**Results:**

We created an annotated Spanish corpus from ProMED-mail health reports regarding disease outbreaks in Latin America. The corpus has been used to train algorithms for two information extraction tasks: named entity recognition and relation extraction. The algorithms, based on deep learning and rules, have been applied to recognize diseases, hosts, and geographical locations where a disease is occurring, among other entities and relations. In addition, an in-depth analysis of micro-average F1 metrics shows the suitability of our approaches for both tasks.

**Conclusions:**

The annotated corpus and algorithms presented could leverage the development of automated tools for extracting information from news and health reports written in Spanish. Moreover, this framework could be useful within EBS systems to support the early detection of Latin American disease outbreaks.

## Background

A broad surveillance strategy to monitor emerging threats to public health involves two components: indicator-based surveillance (IBS) and event-based surveillance (EBS) [1]. A country that uses both, IBS and EBS, is said to be applying epidemic intelligence [2, 3].

IBS, which is based on reports from healthcare providers to public health officials, is a more traditional way of reporting diseases. Its’ main limitation is timeliness. While data provided by IBS systems is essential for early warning and response, it is often late, incomplete, or covers only a limited number of known public health risks. Therefore, emerging or unknown pathogens, as well as rapidly spreading outbreaks or non-reportable events, such as those related to toxic contaminants, may be missed. Thus, with the goal of near real-time detection of infectious disease outbreaks several EBS approaches have been developed in recent years [3]. Particularly, the World Health Organization (WHO) and the Pan American Health Organization (PAHO) state their interest in strengthening EBS systems, in order to detect and respond quickly to all health events and acute risks of any origin, and declare that the capacity to detect and respond to acute public health events must be present at all levels (local, intermediate and national).^1^ Also, the necessity of reinforcing the various mechanisms that perform EBS with the goal of strengthening Global Health Security is stated in the International Health Regulations (IHR).^2^

In EBS, the detection and reporting of signals are often through channels other than traditional surveillance systems. It is usually based on non-official sources, like reports in the media, reports provided by the community, or rumors on the internet and it has been made possible by digital disease surveillance [3]. Hence, EBS systems can be a key component of an effective early warning system, which could enable countries to be well-prepared for endemic and pandemic illness outbreaks. In this sense, event-based surveillance involves fast capturing of data and of early warning signal detection about events that are a potential risk to public health [4]. In particular, during media monitoring, the data can be obtained from several sources such as social media, online newswires, or rumors [4, 5].

In this context, public health surveillance is a natural application of artificial intelligence techniques, mainly in fully automated systems. Moreover, due to the use of social media data, natural language processing approaches are required to extract information.

Much research is being done recently on the use of artificial intelligence in order to enhance event-based surveillance systems with moderated, or partially or fully automated techniques in order to obtain early diagnosis and treatment and to prevent the spread of diseases [2]. However, the use of fully automated techniques has been limited and as far as we know, there is no published research performed in order to extract epidemiological information from Spanish texts. In this article, we provide natural language processing (NLP) resources and techniques in order to enhance digital disease surveillance of diseases prevalent in Latin America based on Spanish texts. The provided resources could be embodied as components of effective early warning systems.

We are particularly interested in monitoring outbreaks of diseases that are prevalent in Latin America. In recent years, several infectious diseases have aroused in this region, for instance dengue, and Zika, which is usually associated with Guillain-Barré syndrome.^3^ Other diseases, such as Chagas (American trypanosomiasis) have existed for many decades. Dengue, Zika and Chagas are part of those diseases named as “neglected (forgotten), tropical and Vector Borne” by PAHO and WHO. They constitute a set of infectious diseases, that primarily affect the most vulnerable populations.^4^ Some endemic diseases, like Hantavirus, appear recurrently in some regions of Argentina and Chile, among other countries. Also, there are currently measles outbreaks in the region (Brazil).^5^ Finally, as it happened with COVID-19, outbreaks of new and also of eliminated diseases could appear.

In order to capture data useful for EBS systems focused on prevalent outbreak diseases in Latin America, we developed information extraction (IE) algorithms to extract named entities (disease, date, geographic location, number of cases, host, origin -reported cause of the disease-, transmission form of the diseases -eg. bite-, modifiers such as negation and uncertainty terms, and terms referring to the past and to conditional expressions) and relations (among the disease -DS- and the geographical location where it occurs, number of cases and the DS, number of cases and geographical location, date and cause of a disease, disease and host to which it occurs, speculation and negation terms and some entities -eg. “the transmission form is suspected to be through ingestion”’-, among others). With the extracted information provided by the named entity recognizers and the relation extraction algorithms, data could be summarized, Moreover, an interface could be constructed, whereby choosing a range of dates and diseases, geographical regions could be visualized on a map (as in HealthMap [6]), highlighting the number of incidences. Also, alerts could be triggered if a number of cases of a given disease surpass predefined thresholds. Other applications of IE from social media through NLP techniques include: the classification of articles and determination of the geographical location of vulnerable populations -both according to threats of particular diseases-, the use of dates to order the appearance of diseases, the use of the number of cases to forecast disease progression [7], and situational awareness and identification of at-risk populations or conditions as an opportunity to enhance public health [8]. In order to develop supervised machine learning algorithms and also to test the developed algorithms, annotated corpora are needed. Since, to our knowledge, there were no Spanish public available corpora, we have constructed an annotated Spanish corpus based on Spanish articles from ProMED-mail [9], a publicly available reporting system for emerging diseases and outbreaks.

Thus, the main contributions of our work are the following:

1. We provide a publicly available Spanish annotated corpus^6^ for named entity recognition and relation extraction of epidemiological information appearing in newspaper articles. The public availability of this corpus contributes to doing open science and to the enhancement of IE techniques by other researchers for extracting information useful for EBS systems. The annotated corpus, contains on one side articles’ titles and on the other hand complete articles, allowing researchers to select the part of the article that best suits their needs,
2. We published the annotation criteria and guidelines used to annotate the corpus [10],^7^ which can help as a guide to other researchers interested in generating their own corpus,
3. We report the techniques developed for automatically extracting information from the articles, and the conclusions of their use on article titles and complete texts. We worked on named entity recognition (NER) and relation extraction (RE), two main IE tasks. Deep learning (a combination of Recurrent Neural Networks -RNNs- and Conditional Random Fields) and a rule-based algorithm were used for NER and a baseline co-occurrence method was used for RE. We report promising results in terms of micro-average F1. The results of the algorithms allow further work with the extracted data. Furthermore, the implementation of the algorithms demonstrates the corpus’ suitability for the task, and
4. We provide an analysis of exact and partial match metrics for NER and of inter-annotator agreement metrics. Usually, not much effort is put into discussing the appropriate methods to measure the performance of NER systems, nor are provided the implementation details of inter-agreement metrics for NER tasks. The use of appropriate metrics is crucial for algorithm results validation in a useful way.

We consider that the resources and analysis provided contribute to the growing field of the use of artificial intelligence to enhance EBS systems.

The rest of the paper is organized as follows. “Related work” section presents previous work in event-based surveillance systems in public health. In “Methods” section we describe the creation of our annotated Spanish corpus along with its challenges and their solution, the information extraction methods developed to perform NER and RE, and the evaluation methods used. “Results” section shows results obtained from the IE techniques proposed. In “Discussion” section, we perform error analysis and discuss the obtained results and the implications of the findings in the context of existing research and present limitations of this study. Finally, “Conclusion” section states the main conclusions and explains the relevance of the study to the field. Last, Declarations are presented.

## Related work

Research studies show that social media may be a valuable data source in disease surveillance systems aimed to detect disease outbreaks, since it helps information to be reported faster than traditional methods. Therefore, social media allows the enhancement of outbreak response [11], and its use and the use of monitoring techniques to support surveillance of potential health threats activities has grown over the last years [5, 12]. For example, Twitter data has been used to several health surveillance tasks evidencing good results [13]. Furthermore, O’Shea [2] described 50 event-based Internet systems, in 99 articles.

According to Zeng et al. [4], internet-based surveillance systems can be organized into three types: moderated, partially moderated, and fully automated regarding the amount of manual effort to provide information. For instance, the Program for Monitoring Emerging Diseases (ProMED), an internet-based reporting system dedicated to the dissemination of information on epidemics of infectious diseases that depends on the International Society for Infectious Diseases (ISID), is a moderated system, that could benefit regions and countries in complementing their undiagnosed disease surveillance systems [14].

It is worth mentioning the effectiveness of using ProMED data to deal with coronaviruses [15]. Other moderated systems are epidemiological bulletins issued by different regions or countries.^8^ On the other hand, the Global Public Health Intelligence Network (GPHIN), an event-based multilingual early-warning and situational awareness network for potential public health threats worldwide, is a partially moderated system developed by the Canadian Government. GPHIN detected severe acute respiratory syndrome (SARS) more than two months before the first publications by the WHO [2]. Finally, examples of fully automated systems include the European Commission’s Medical Information System (MedISys),^9^ Pattern-based Understanding and Learning System (PULS),^10^ SENTINEL,^11^ DEFENDER [16] and the Global Rapid Identification Tool System (GRITS) [17].^12^

It is worth noting the Epidemic Intelligence from Open Sources (EIOS) initiative [17], a collaboration of WHO, the European Union, and several organizations, that pulls together many existing aggregator systems, as those aforementioned, with the aim to unify health approaches to digital surveillance from public sources.

Next, we list the works more related to ours. They address the same objective of extracting detailed information from social media; however, they used different techniques and are meant for English, while ours is for Spanish.

EventEpi [17] uses data from WHO Disease Outbreak News and ProMED-mail, and automatically extracts information regarding the disease, country, date, and confirmed-case count. To do so, they performed NER in two steps: EpiTator, an open-source epidemiological annotation tool, suggested many different possibilities for each entity, so they extracted the key country and disease using a heuristic. In the second step, they trained a Naive Bayes classifier to find the key date and confirmed-case count. Another closely related work is the Platform for Automated extraction of Disease Information from the web (PADI-web) [18]. This tool generates epidemiological information on diseases, locations, dates, hosts, and the number of cases for outbreaks mentioned in the news and social media articles. Therefore, they use rule-based systems, data mining techniques and a support vector machine trained to better classify or identify information on diseases.

Finally, different challenges have been organized lately for named entity recognition and relation extraction in Spanish biomedical texts [19, 20]. Although they are focused on a different domain (biomedical texts instead of newspaper articles), the use of advanced techniques used by the participants for performing NER and RE in Spanish texts can be appreciated.

## Methods

In this section, we provide details and describe the challenges of the task of creating an annotated Spanish corpus. In order to do that, we describe how we selected the dataset, the data cleansing process performed, the annotation schema and guidelines, the ethic statements and the annotation process. We also show the dataset analysis and the inter-annotator agreement. After, we present the information extraction methods developed to perform NER and RE tasks (a rule-based and a machine learning method for NER and a co-occurrence method for RE). Finally, we describe the evaluation methods used.

### Creation of an annotated Spanish Corpus

Given that, as far as we know, there was no publicly available corpus for this task, we decided to create an annotated corpus. This corpus and its subsequent publication would enable us to train machine learning algorithms to detect named entities and extract relations from Spanish texts. Moreover, it could also help advance the task, given that other researchers could use it to improve the algorithms used for extracting information useful for EBS systems. The creation of the corpus has many challenges: entities and relations have to be defined based on the questions that would like to be answered (there is no standard way of defining entities and relations), annotators have to be selected and trained (the training involves many rounds of training and annotating, and it is a process that is highly time-consuming. Besides, the fact that the annotation effort was not financially compensated, makes the task more difficult), a criteria has to be established to select and clean the dataset, and then this task has to be performed. Finally, there are many ways of implementing the chosen inter-annotator agreement metric, so the advantages and disadvantages of each one had to be discussed. In order to face the challenges, we developed an iterative process of creating and documenting the annotation schema and criteria, training the annotators, and measuring the inter-annotator agreement. We executed the process until we obtained a stable (good inter-annotator agreement, few doubts) annotated dataset. In this subsection, we present the process of creation of the corpus, including ethic and data statements [21] and an analysis of the resulting annotated dataset.

#### Dataset selection

In order to construct the corpus we downloaded articles from ProMED-mail, a reporting system dedicated to the rapid dissemination of information on epidemics of infectious diseases, among others [9].^13^ The articles published on ProMED-mail have been edited based on journalistic notes from different media by an interdisciplinary staff.

**Fig. 1:**
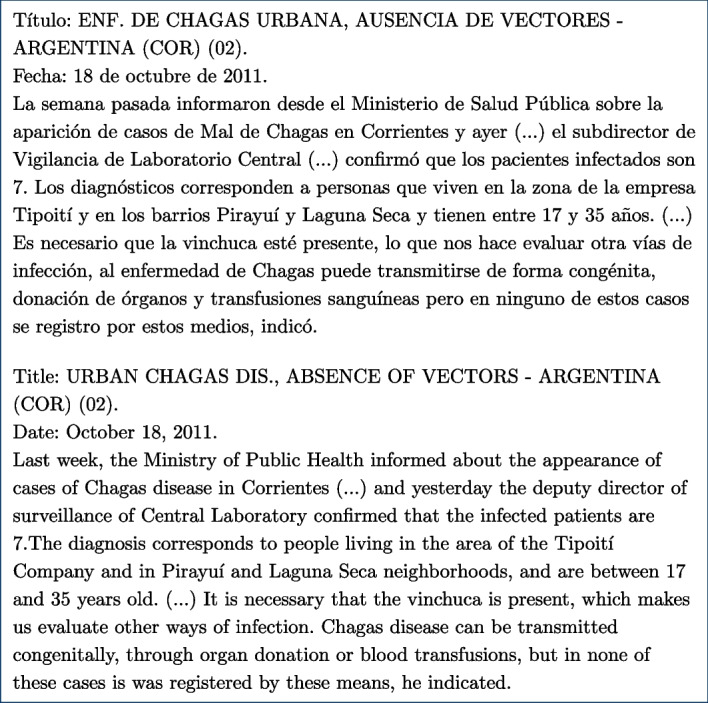
Example of a ProMED-mail article without the associated metadata and its’ translation to English

ProMED-mail articles are formed by a title, a date, the main text, and metadata (such as source and editor of the articles). For an example, see Fig. 1.

Since titles of the articles are informative and easier to annotate than whole articles, we decided to work with: (1) only the title and the date of the article (from now on *the Title*), and (2) the main text of the article -including the title, the date and excluding the metadata- (from now on *PMA* -ProMed mail article-). Based on the results of working with both parts of the articles, we expect to be able to determine which is the best selection for future works.

We retrieved 1377 articles written in Spanish and focused on reported issues in Latin American Spanish and Portuguese-speaking countries that mention the appearance of at least one of the following pathologies: dengue, hantavirus, measles, Guillain-Barré syndrome, Zika and Chagas. The retrieved articles were written between 23 August 2001 and 18 August 2020.

#### Data cleansing

We cleansed the data by (1) removing metadata with the use of regular expressions and (2) performing data normalization (unification: of the date separator character -dashes and slashes are used-, of the date format -March 12 2012 and March 12, 2012 are used-, of the decimal separator in numbers -semicolons and dots are used-, and of the name of countries -acronyms were transformed to full names-, among others).

#### Annotation schema and guidelines

Seven named entities (disease -DS-, date -DT-, location -LOC-, number of cases -NoC-, Origin -OR-, host -Hst-, and transmission form -TF-) and three modifiers ( past terms -PT-, negation -NT-, and uncertainty -UT-) have been annotated. Besides, binary and ternary relations among the named entities and the modifiers were annotated.

The main annotation guidelines include: (1) only hosts with a special characteristic should be annotated (eg. pregnant women, infants and dogs), (2) hosts are the carriers of the disease (not those of a secondary virus that causes the disease), (3) negations and speculations should be annotated only if related to a named entity or to a relation, (4) the largest possible term has to be annotated, eg. “virus de sarampión D8” should be annotated rather than “sarampión” in “virus de sarampión D8” (*measles virus D8*), (5) relations between sentences should not be annotated, and (6) misspelled terms corresponding to named entities should also be annotated.

For more detailed information about the annotation schema and guidelines please refer to our previous work Dellanzo et al. [10].

#### Ethic and data statements

Bender and Friedman proposed “data statements” [21], which address biases and other critical issues that emerge when working with natural language. In the following paragraphs we describe the ethic and data statements of our corpus.

As aforementioned, we selected Spanish ProMed-mail texts, describing cases of infectious diseases in Spanish and Portuguese-speaking Latin American countries, that mention at least one of the following terms: chagas, measles, hantavirus, Guillain Barré or Zika, and that were written between 23 August 2001 and 18 August 2020.

The type of language used is the usual in newspaper articles. ProMED articles are a shortened version of the original articles. Although each Spanish-speaking Latin American country uses the language in different ways (e.g. often different terms are applied to the same objects), in the articles, standard Spanish is used. There is no information available of ProMed-mail editors’ demographics.

The annotation was carried out by eight Spanish native speakers from Peru and Argentina, where different variations of Spanish are spoken. Nevertheless, we evaluate that this fact did not hinder an accurate understanding of the annotation criteria or of ProMED-mail articles. The annotation team was composed of five computer science master students, one linguist, and two PhDs in computer science, researchers in natural language processing and with experience developing annotation criteria and annotating in different domains (from now on, “the experts”). Annotators were not economically compensated.

#### Annotation process

The Model-Annotate-Model-Annotate (MAMA) cycle [22] was followed to do the annotation process.

After writing a document with the annotation schema and guidelines and discussing it with the annotators an annotation round was performed. A portion of the articles was annotated not only by regular annotators but also by one of the annotation experts. After a revision of doubts and differences in the annotation criteria, the annotation guidelines were refined and new articles were annotated. After three annotation-revision iterations, the final guidelines were defined and annotations were performed. The entire annotation process took approximately 15 months. Disagreements were solved by the experts.

The annotation was done with the brat rapid annotation tool [23].

#### Dataset analysis

Overall 513 different articles have been annotated. The average number of sentences and words per article is 10 and 334 respectively. The average number of words in titles is 10. A total of 11, 193 (3789 different) entities and 6679 (4776 different) relations have been annotated.

After performing the annotation, we analyzed the resulting dataset in order to know the number of existing entities and relations. The analysis of the annotations is calculated for the whole set of 513 annotated titles and PMAs. Table 1 shows the number of total and different entities and their modifiers for titles and for PMAs, respectively, whereas Table 2 shows the same for relations, and includes entities related by each relation.

**Table 1:** Type and amount of total and different entities and modifiers annotated for Titles and PMAs

Entity/modifier	Titles		PMAs	
	Total	Diff.	Total	Diff.
Date (DT)	491	462	613	550
Disease (DS)	560	35	3053	229
Host (Hst)	33	19	678	357
Location (LOC)	573	189	2611	884
Number of cases (NoC)	10	5	2176	758
Origin (OR)	47	21	778	270
Transmission form (TF)	30	15	293	168
Negation (NT)	3	2	74	37
Past (PT)	1	1	504	328
Uncertainty (UT)	17	8	400	197

**Table 2:** Relations annotated among entities in articles’ titles and PMAs

Relation	Entities	Titles		PMAs	
		Total	Diff.	Total	Diff.
DsOccIn	DS-LOC	573	254	1838	924
DtDs	DT-DS			89	75
NegDs	NT-DS	1	1	30	28
NegNoC	NT-NoC			28	26
NegOr	NT-OR	2	1	18	16
NegTf	NT-TF			13	12
NoCDs	NoC-DS	8	5	1164	817
NoCHst	NoC-Hst			214	198
NoCLoc	NoC-LOC	4	4	810	773
OccTo	DS-Hst	36	26	408	254
OrDs	OR-DS	38	25	596	300
OrOccIn	OR-LOC	14	13	66	60
PtDs	PT-DS			203	189
PtNoC	PT-NoC			389	379
TfDs	TF-DS	14	8	105	77
TfOccIn	TF-LOC	7	6	18	17
TfOr	TF-OR	8	6	149	134
UcDs	UT-DS	6	5	89	75
UcNoC	UT-NoC			271	249
UcOr	UT-OR	5	5	58	54
UcTf	UT-TF	3	3	18	17
UcNoCDs	UcNoCDs-NoC-DS			60	59
UcOrDs	UcTfOr-DS-OR	4	4	36	36
UcTfDs	UcTfDs-DS-TF	3	3	5	5

It can be seen that 92% of the entities annotated in Titles are of type “date”, “disease” and “location”, and 79% of Titles’ relations are of type “DsOccIn” (among diseases and locations). Other entities (such as “transmission form” or “origin”) and relations can only be seen in PMAs.

There are multiple factors that influence the number of different diseases annotated, even though the scope in the annotation guidelines was reduced: (1) the existence of spelling errors (eg. “Zica” instead of Zika and “gullain barre” instead of Guillain-Barre), (2) different ways to refer to the same illness or reference to different serotypes of dengue (eg. “DEN-1” and “DEN-4”), (3) the annotation by mistake of conditions or diseases that were not under the scope of our study (eg. “blindness”, “glaucoma” and “pneumonia”), and (4) the annotation of entities as diseases in order to be able to annotate a relation with other entities (eg. the words “disease”, “virus” and “infection”). Other annotation mistakes include: (1) the difficulty to differentiate some entities (eg. origin vs. transmission form, and date vs. past), 2) differences in criteria regarding the span of entities (eg. “pregnant woman” vs. “pregnant”, and “suspected cases” vs “cases”), (3) differences in criteria regarding joint vs different entities (eg. “Brasil (Sao Paulo)” vs. “Brasil” and “(Sao Paulo)”, and (4) the fact that some entities not co-occurring with others were annotated (eg. date).

The relations not found in titles are the date in which a disease is found (DtDs) (this is because the date and the rest of the title are in different sentences and relations among sentences are not annotated), negation of transmission form (NegTf), the uncertainty regarding number of cases and number of cases of a disease (UcNoc and UcNocDs), the number of cases of a disease for a particular host and a past term related with disease and with number of cases (PtDs and PtNoC).

#### Inter-annotator agreement

The inter-annotator agreement (IAA), useful to evaluate the consistency among the annotations, was calculated using Cohen’s Kappa coefficient $\kappa$ [24]. We implemented $\kappa$ with the scikit-learn library,^14^ in which given two arrays (each corresponding to the annotation of a different annotator), that contain the labels assigned for each token (entities, and binaries and ternaries relations), returns the corresponding score for that annotated article. One token can have multiple labels associated, whether it’s an entity or a relation (eg. Zika can be a disease or a cause). In our $\kappa$ implementation, we consider that one token was annotated equally by two annotators if and only if both annotators assigned the exact same set of labels to it (or no labels at all). That is, if there is a partial coincidence it will be counted as if there were no coincidence at all.

Table 3 shows the IAA for the three first annotation rounds. It can be appreciated that it improves in the second iteration but worsens in the third. In our opinion, this is due to the fact that the annotation task was voluntary (without payment). Besides, the annotation-revision iterations, for which the IAA was measured, took six months, so some of the first annotators were not able to continue annotating, and new annotators had to be contacted and trained for further annotation rounds. Finally, in the third iteration there were fewer articles annotated by more than one annotator.

**Table 3:** Kappa results for each iteration

Iteration	# Annotated articles in common	$\kappa$
1	22	0.48
2	16	0.62
3	7	0.49

**Fig. 2:**
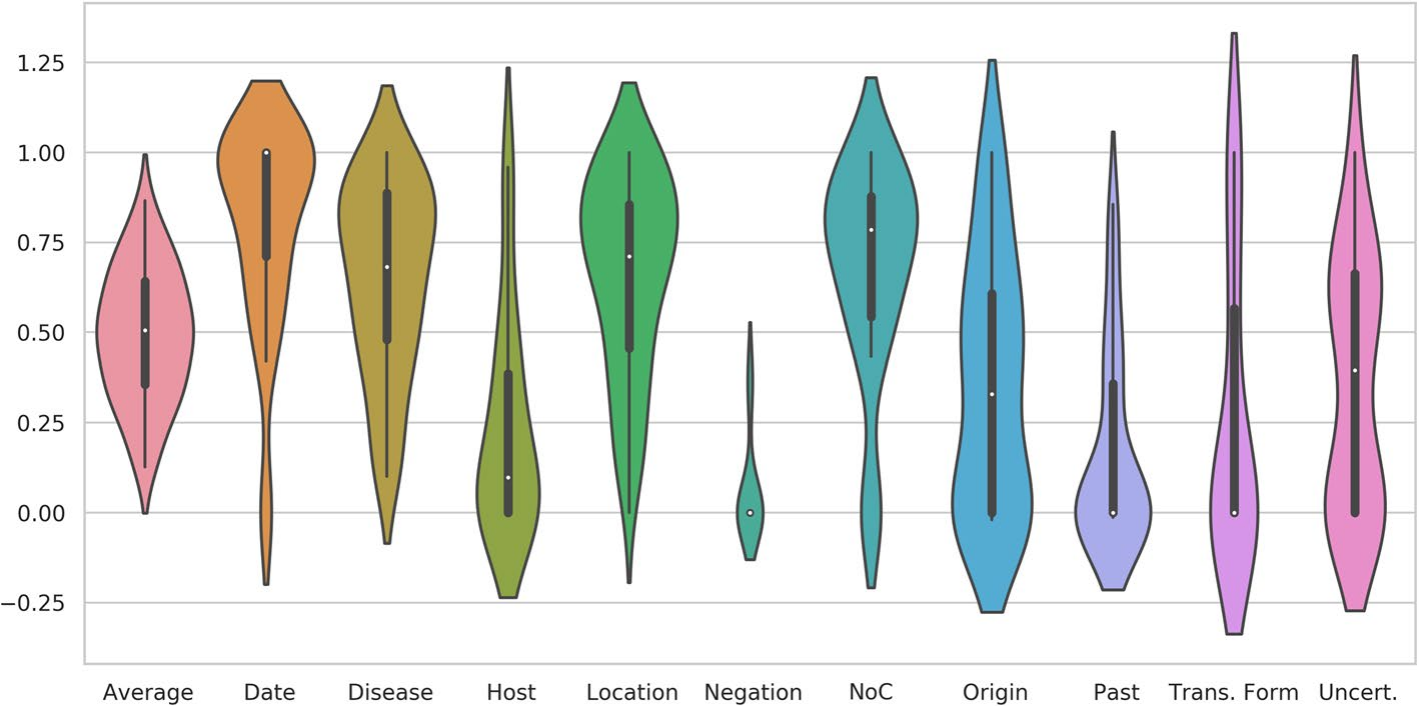
Violin chart of each entities’ Cohen’s Kappa value and average Cohen Kappa

Figure 2 shows a violin chart, where the kappa-value-average of all annotated articles per entity can be seen. Also, the average for all entities is shown. This analysis was done for those PMA articles annotated by more than one annotator (those referenced in Table 3).

The width of each figure in the violin chart represents the number of entries for that $\kappa$ value. The black box inside the violins is a box plot. It shows the interquartile range (IQR), which is the distance between the first and third quartile. The white dot inside it represents the median over all the data set. The first quartile (bottom of the box), indicates the median of the lower half of the data, whereas the third one (top of the box) represents the median of the upper half. The lines extending from the box (called whiskers), show the minimum and maximum adjacent values. Any value that is not within the scope of the box and whiskers, is considered an outlier.

It can be noticed, that entities with better $\kappa$ are “date”, “disease”, “location” and “number of cases”, which were the most simple ones. On the other hand, the entities with lower results are those that were harder to define.

### Information extraction

Once we have defined the annotated corpus, this can be used for training information extraction algorithms. In particular, in this section, we present the methods developed to perform name entity recognition and relation extraction. For more information about the methods, please refer to [25]^15^ and [10].

#### Named entity recognition

The aim of NER algorithms is to automatically extract entities from articles -titles and PMAs- and classify them according to their type (such as date and disease). To do so, we developed a rule-based method and a machine learning approach.

### Rule-based method

In this method, rules were developed based on the analysis of patterns present in thirty articles separated in a stratified manner by disease and that were later not used for the test set.

Candidate **number of cases (NoC)** were detected by the use of Freeling [26]. Then, numbers and ordinal adjectives: (1) containing numbers and eventually dots or commas, (2) not followed by certain terms (eg. *day*, *month*, *year*, *percentage*), and (3) at a maximum distance of seven words to a list of terms that might be related to number of cases (eg. *cases* and *infected*) were tagged as NoC.

In order to tag **locations (LOC)**, we created a Gazetteer with *GeoNames*^16^ data including Latin American cities with more than 5000 inhabitants and larger cities from the rest of the world, regions, countries, and continents. From the tokens tagged as location by Freelings’ named entity recognition and named entity classification modules, only those belonging to Latin American Spanish or Portuguese-speaking countries were kept.^17^ In PMAs locations were tagged only if they are related to the location mentioned in the title (eg. cities belonging to a country) and if they co-occur in the same sentence as a NoC entity. In case of ambiguities, the location with the highest population was chosen.

The rest of the named entities (**diseases**, **hosts**, **origin**, **transmission form**) and modifiers (**negation**, **uncertainty**, and **past terms**) were detected by the use of regular expressions and lists of terms, developed based on the analysis of the articles separated for this purpose. As mentioned in [10], dates, negation, uncertainty, and past terms were recognized as named entities only if they co-occur in the same sentence and within a fixed distance to another entity to which they might be related.

### Machine learning method

We also developed a connectionist approach for NER based on Flair [27], which combines Recurrent Neural Networks (RNNs) and Conditional Random Fields.

Recurrent Neural Networks are powerful dynamic systems that process an input sequence one element at a time ($x_t$), maintaining in their hidden units ($h_t$) a state vector that implicitly contains information about the history of all past elements of the sequence. In the context of NER, a sequence of words ($x_1, \ldots , x_t,\dots x_n$) can be encoded using hidden units ($h_1, \ldots , h_t,\dots , h_n$). Each hidden state $h_t$ encodes information about the current word ($x_t$) and its previous sequence ($h_{t-1}$) that eventually allows to predict its entity label ($\hat{y}_t$). The hidden state $h_t$, can be obtained as follows:

$$h_t= {\left\{ \begin{array}{ll} \sigma (W_{xh}x_t+W_{hh}h_{t-1} + b_h), &{} t\ge 1 \\ 0, &{} t = 0 \end{array}\right. }$$

where $W_{xh}$ denotes the weight matrix connecting $x_t$ to $h_t$, $W_{hh}$ denotes the weight matrix connecting $h_{t-1}$ to $h_t$, and $b_h$ is the bias. $\sigma$ is a non-linear function. It is worth noting the contribution of $h_{t-1}$ (past memory) to define $h_t$. Once we have defined $h_t$, we can use it to predict the entity label as follows:

$$\begin{aligned} \hat{y}_t = \text{ softmax }(W_{hy} h_t+b_y) \end{aligned}$$

where $W_{hy}$ denotes the weight matrix connecting $h_t$ to the set of possible entity labels, and $b_y$ is the bias. To avoid vanishing gradient issues, vanilla RNN units ($h_t$) are usually replaced by gated recurrent units such as Long short-term memory (LSTM), which are well-suited to remember values over long sequences.

On the other hand, Conditional Random Fields (CRFs) are probabilistic models that allow to compute the probability *p*(*y*|*x*) of a possible output, i.e., the probability of the entity labels in NER ($y_1,\ldots , y_n$), given the input of words ($x_1,\ldots ,x_n$).

The conditional probability *p*(*y*|*x*) can be written as follows:

$$\begin{array}{rcl} P(y|x) &{} = &{} \frac{p(x,y)}{p(x)}\\ &{} = &{} \frac{p(x,y)}{\sum _y p(x,y)} \\ &{} = &{} \frac{\frac{1}{Z}\prod _{C \in \mathcal {C}} \Phi _C(x_C,y_C)}{\frac{1}{Z} \sum _y \prod _{C \in \mathcal {C}} \Phi _C(x_C,y_C)} \end{array}$$

From this, the general model formulation of CRFs is derived:

$$p(y|x) = \frac{1}{Z(x)} \prod _{C\in \mathcal {C}} \Phi _C (x_C,y_C)$$

where $\Phi _C$ are the different factors corresponding to maximal cliques in the independency graph of probabilistic variables. Each factor corresponds to a potential function that combines different features $f_i$ of the considered part of the observation (words) and the output (entity labels). The normalization *Z* is defined as follows:

$$Z(x) = \sum _{y'}\prod _{C\in \mathcal {C}} \Phi _C (X_C,y')$$

where $y'$ is a variable to be marginalized by summing out their values. Usually, CRFs are used on top of RNNs to improve the classification task. Thus, each $h_t$ is considered a potential function $f_i$ which allows to compute $p(\text{ labels }|\text{ sequence})$.

We implemented a bidirectional recurrent neural network (BiLSTM), and a subsequent Conditional Random Fields decoding layer with contextual string *embeddings*.

The implementation was made using the library provided by Flair’s authors. For training, we initialized the sequence tagger with Spanish word embeddings (value vectors that encode semantics similarity among words) and trained the model with a portion of the annotated corpus (training dataset) with the chosen parameters.

We performed a stratified (based on disease) development (train)-test split (80–20%). With the development dataset, we did stratified (also by disease) five-fold cross-validation for hyperparameters optimization. The selection of parameters was based on the one suggested by Reimers et al. [28] for NER. For each experiment, we fixed all parameters but one and tested different values for this parameter. Once selected the best value, we fixed this value and tested -in the same way- a different parameter. Below we describe the parameters and values tested. Those in bold are the ones we chose because with them we obtained better results.

- Optimizer: **Stochastic Gradient Descent (SGD)** and Adam [29], each one with different learning rates: [0.001, 0.05, 0.01, **0.1**].
- Mini batch size: [**8**, 16, 32].
- Recurrent neuronal network layers: [1, 2, **3**].
- Hidden size: [80, 128, **256**].
- Variational dropout: [0.00, 0.05, 0.1, **0.5**].
- Embeddings: we analyzed seven different combinations of embeddings using the Spanish Billion Word Corpus (SBWC)^18^ [30] and Wikipedia dumps (retrieved from the Flair library^19^) through *stacked embeddings*. The best embedding’s configuration was a stacked embedding consisting of *SBWC-FastText* and Flair embeddings (in its backward and forward implementation).

Finally, the neural network was trained with the development set with the best-found parameters and tested with the test set. The same test or heldout dataset was used to evaluate results of the rule-based algorithm.

#### Relation extraction

The rules for the relation extraction algorithm were elaborated based on the analysis of 63 articles. The method was tested with 450 articles (all annotated articles not used to perform the analysis).

We developed a baseline relation extraction algorithm, that detects as relations those pairs or triples of entities that co-occur in the same sentence and that have less than a pre-defined distance between them and that belong to the type of possible relations defined in the annotation schema. Possible relations among the same token are not considered (eg. Zika can be a disease and a cause). For the algorithm, we used as named entities those annotated by the annotators instead of those discovered by our NER algorithms. This was necessary in order to be able to evaluate the relations discovered by the algorithm with respect to our gold standard.

The maximum distance allowed was defined based on the analysis of the 63 previously separated articles and it depends on whether one of the entities involved is a modifier or not. If so, we took 50 characters -approximately 10 words-20 as maximum distance. Otherwise 100 characters -approximately 20 words- were taken.

#### Evaluation methods

We evaluate our NER and RE methods by using Precision, Recall and F1 metrics.

For NER we used an exact match as well as a partial match. In the first case, a match is accounted as True Positive if and only if the exact same tokens are tagged by the NER algorithm and by an annotator as the same type of entity. In the second case, an overlap is also taken into account as long as it has the same entity type. For partial match we slightly modified our implementation [31], that was based on Message Understanding Conference’s (MUC) partial match [32].

Our relation extraction algorithm is evaluated with precision, recall and F1 based on exact match. True positive, false positive and false negative values are calculated based on the existence of a relationship among the entities in the annotated dataset.

## Results

Tables 4 and 5 show results for the name entity recognition task for each entity in Titles and PMAs for the rule-based and machine learning methods.^21^ Exact and partial micro-averaged F1-score results are shown.

**Table 4:** Results of rule-based and RNN methods for NER detection in Titles

Entity/modifier	Rule-based		RNN	
	Exact match	Partial match	Exact match	Partial match
Date	0.73	0.87	0.94	0.96
Disease	0.96	0.97	0.94	0.96
Host	0.82	0.88	0.20	0.20
Location	0.62	0.77	0.67	0.84
Number of cases	0.19	0.20	0.00	0.00
Origin	0.13	0.11	0.33	0.27
Transmission form	0.22	0.50	0.36	0.57
Negation	0.00	0.00	–	–
Past	0.00	0.00	–	–
Uncertainty	0.44	0.44	0.00	0.00
Micro-average	0.73	0.82	0.81	0.89

F1 is shown per entity type for exact and for partial match. Also micro-averaged F1 is shown

**Table 5:** Results of rule-based and RNN methods for NER detection in PMAs

Entity/modifier	Rule-based		RNN	
	Exact match	Partial match	Exact match	Partial match
Date	0.51	0.62	0.75	0.73
Disease	0.75	0.77	0.73	0.76
Host	0.31	0.40	0.53	0.60
Location	0.51	0.56	0.63	0.68
Number of cases	0.52	0.53	0.74	0.75
Origin	0.36	0.45	0.47	0.50
Transmission form	0.14	0.21	0.24	0.40
Negation	0.14	0.17	0.10	0.11
Past	0.18	0.26	0.36	0.49
Uncertainty	0.21	0.31	0.33	0.43
Micro-average	0.52	0.56	0.64	0.68

F1 is shown per entity type for exact and for partial match. Also micro-averaged F1 is shown

Table 6 shows precision, recall and F1 from the relation extraction algorithm for each relation and the micro-averaged results for Titles and PMAs.

**Table 6:** Results of relation detection algorithm for Titles and PMAs

Relation	Titles			PMAs		
	P	R	F1	P	R	F1
DsOccIn	0.93	1.00	0.97	0.89	0.89	0.89
DtDs				0.92	0.87	0.90
NegDs	0.00	0.00	0.00	0.51	0.95	0.67
NegNoC				0.42	1.00	0.60
NegOr	1.00	1.00	1.00	0.75	1.00	0.86
NegTf				0.57	1.00	0.72
NoCDs	0.88	1.00	0.93	0.82	0.91	0.86
NoCHst				0.48	0.97	0.64
NoCLoc	0.67	1.00	0.80	0.33	0.91	0.48
OccTo	0.89	1.00	0.94	0.83	0.88	0.86
OrDs	0.79	1.00	0.89	0.87	0.86	0.86
OrOccIn	0.33	1.00	0.50	0.30	0.76	0.43
PtDs				0.56	0.92	0.70
PtNoC				0.67	0.93	0.78
TfDs	0.65	1.00	0.79	0.62	0.82	0.71
TfOccIn	0.44	1.00	0.61	0.31	1.00	0.47
TfOr	0.50	1.00	0.67	0.76	0.93	0.84
UcDs	0.45	1.00	0.62	0.29	0.96	0.45
UcNoC				0.57	0.98	0.72
UcOr	1.00	1.00	1.00	0.76	0.96	0.85
UcTf	0.50	1.00	0.67	0.75	0.90	0.82
UcNoCDs				0.29	0.43	0.35
UcOrDs	0.33	0.33	0.33	0.27	0.31	0.29
UcTfDs	0.00	0.00	0.00	0.00	0.00	0.00
Micro-avg	0.86	1.00	0.92	0.62	0.90	0.73

Binary and ternary relations’ Precision (P), recall (R) and F1-score are shown. Also micro-averaged metrics are shown

## Discussion

In this section we will discuss results of our algorithms, implications of the findings in context of existing research, and limitations of our study.

### Named entity recognition

In this subsection we will analyze and discuss the results of NER algorithms presented in Tables 4 and 5, that show exact and partial match F1 for NER with both methods for Titles and for complete articles.

It can be noticed that “date”, “disease” and “location” entities are easier to detect in Titles than in the whole text (better F1 in exact and partial match). Although there are fewer entities of these types in Titles than in PMAs (see Table 1), as working with titles is less time-consuming than working with the main texts (PMAs), we conclude that if “date”, “disease” or “location” are to be recognized, it would arguably be better to do so from Titles than from the whole texts. Besides, those are the most common entities found in articles’ titles (92% of the entities are of these types). On the other hand, the number of cases has much better results in complete articles, since not many NoCs appear in titles. Furthermore, regarding the main texts, the entities with better F1, both in exact and partial match, are “date”, “disease”, “location” and “number of cases”.

As could be imagined, in almost all cases partial match has better results than exact match. In Titles, “disease”, “number of cases”, “origin” and “uncertainty” with the rule-based algorithm and “host” with the RNN are the exceptions. The reasons are the following: (1) Only 4.5% terms corresponding to diseases are composed of more than one word. From them, only one had a partial match with each of the methods, (2) there are only two annotated NoCs, (3) the different results for origin’s exact and partial match are given by the different formulas used for calculating the metrics, and (4) the only match for the Host entity is from a one word term. In PMAs, for the rule-based method disease and NoC have similar exact and partial matches, for the RNN method dates, NoC and negation also have similar F1s. This is due to the fact that in almost all cases these entities are composed of one-word terms.

Beyond these cases, in our opinion, partial match is adequate and gives a better notion of the results of the algorithms than exact match, since also matchings among annotations of different annotators are also sometimes partial (eg. “municipio de Lábrea, en el sur del Estado Amazonas” -Municipality of Labrea, in southern Amazonas State- vs “sur del Estado Amazonas” -southern Amazonas State- and “síndrome pulmonar por hantavirus (SPH)” -hantavirus pulmonary syndrome (HPS)- vs hantavirus).

#### Error analysis

Below we analyze some F1 values, and also precision and recall (not shown in Tables 4 and 5). Regarding Titles (Table 4), for the rule-based algorithm NoCs’ F1 is low (0.19). This is because there are only two annotated entities of this type. The method detects both (recall -R- 1), but also other terms not annotated by mistake (precision -P- 0.11). Regarding “origin”, there are only 11 terms annotated as such. The RNN method detects only 2 (that are in fact of this type). Therefore, P is 1 and R is 0.2.^22^ Besides, there are 13 uncertainty terms in the training dataset and 3 in the test dataset (that were not discovered). Therefore UT F1 is 0 with the RNN method.

The RNN algorithm obtained better micro-averaged F1 than the rule-based method for both exact and partial matches and for titles and for complete articles. Furthermore, the RNN algorithm has some advantages over the rule-based: (1) it can detect more variations of entities (eg. more ways to refer to a certain disease or different serotypes of the virus that causes dengue), (2) it recognizes different formats to refer to dates and locations (eg. the date “12 December, 2012” is labeled as two different dates by the rule-based method, but correctly as one by the RNN algorithm), and (3) it detects non-numeric “NoCs” (eg. “10 cases per 100.000 people”). On the other hand, the rule-based method has some advantages over the RNN method: (1) it detects all types of entities (in the training set of the RNN, some entities had zero appearances, hence the algorithm is not able to detect them),^23^ and (2) it correctly labels “Zika” as a cause of microcephaly when the RNN method does not. Other disadvantages of the neural network approach, are the following: (3) it detects “NoC” of diseases not of interest (eg. cases of hepatitis), (4) it labels all dates found in the body of the articles as “Past” instead of as “Date”, even when it’s the same date as the one in the title, and (5) as expected, it carries annotation errors (eg. it labels diseases not of interest as “syphilis”, which were mistakenly labeled by the annotators). Finally, the connectionist approach needs an annotation effort, while the rule-based method requires the elaboration of rules.

In the case of the rule-based algorithm, those entities that involved more complex rules to be recognized (Date, Location and Number of cases) had better results than the others.

Last, we found some annotation errors (eg. dates annotated as “Date”, when they should have been annotated as past, difficulty in differentiating “Transmission Form” and “Origin”, and lack of annotation of some entities).

### Relation extraction

In this subsection, we will analyze the results of the relation extraction algorithm that can be seen in Table 6. The entities related by each relation can be seen in Table 2 and their expanded names in Table 1.

Those relations that do not exist in Titles (i.e. the annotators didn’t detect any relation of certain types in the annotated dataset) , appear with no results in Table 6. In those found, although it can be noticed that in almost all cases relations have better results for articles’ titles than for PMAs, in general terms we can conclude that the interesting results are those found in PMAs, since there are very few relations in Titles (for more information see Table 2). Nevertheless, the relation “DsOccIn”, that indicates in which geographical location a disease is found has 0.97 F1 in Titles and 0.89 F1 in PMAs (with 573 and 1838 annotated relations in the whole dataset respectively).

This relation constitutes 79% of the relations of the entire corpus and 76% of the training dataset and it is interesting to notice that from the analysis of Titles we can automatically extract where a disease is located with very good results, that are even better than those obtained in PMAs.

The relations with better F1 (F1 above 0.82 and with more than 89 appearances) in PMAs are: the geographical location of the disease (DsOccIn -0.89 F1-), the date where a disease is informed (DtDs, -0.90 F1-), the number of cases of a disease (NocDs -0.86 F1-), to which host it occurs (OccTo -0.86 F1-), the cause of the disease (OrDs -0.86 F1-), and the relation among a transmission form and a cause (TfOr) -0.84 F1- (eg. in “[Zika] (Or) virus is transmitted mainly by [mosquitoes] (Tf)”, there is a “TfOr” relation among the two entities.

Based on these results, we can conclude that if we only want to detect the diseases and where they are occurring it suffices to analyze Titles. In order to obtain more information, automatic relation extraction from PMAs should be carried out. Besides, for those relations found in Titles, recall is almost always 1 and is higher than precision. In many cases, this is because there were few annotated entities and all of them were found by the algorithm, but the algorithm discovered also many other relations that were not annotated (low precision). This is probably due to the fact that the algorithm annotates all relations found, given that the restrictions related with the distance usually do not apply in titles, because of their short length. As an example, consider the following title with tagged entities “ENFERMEDAD DE [CHAGAS] (DS) - [VENEZUELA:A (MER)](LOC) (02) [TRANSMISIÓN ORAL](TF), BROTE FAMILIAR.” “(CHAGAS DISEASE - VENEZUELA (MER) (02) ORAL TRANSMISSION, FAMILY OUTBREAK.”) which has a relation of type “TfOccIn” from “TRANSMISION ORAL” to “VENEZUELA”. In this case, there were 7 instances of the relation annotated. All of them were found by the algorithm (recall 1), but 16 were discovered (precision 0.44). Therefore, the algorithm should be improved in order to increase precision. One way to do it is to only tag as relations those, whose related entities when co-occurring in the analysis dataset, are related in more than 50% of the cases.

Analyzing results from both NER methods shown in Table 5 and inter-annotator agreement, shown in Fig. 2, it can be noticed that those entities with lower IAA do not have good results with any of the methods. Nevertheless, those entities with better IAA have better results with both methods. This works as expected since entities not easily distinguishable by annotators are probably not recognized by automatic methods (in the case of the NN because it learns from the data, and in the case of the rule-based algorithm, because rules followed by the data may not be clear). Besides, the definition of those entities with low IAA should be reviewed.

As mentioned earlier, there are previous works related to ours. Nevertheless, they use different datasets (each work built its own corpus), they are for English and they use different techniques. EventEpi [17] also automatically extracts information from ProMED-mail (English) reports. Therefore, they train a Naive Bayes classifier. While our approach also uses ProMED-mail, we focus on creating a sound corpus which eventually is used for NER and RE using a deep learning algorithm and also rule-based algorithms as baselines. Besides, we work for Spanish. On the other hand, unlike our techniques which are based on deep learning, PADI-web [18] combines information extraction based on rule-based systems, data mining techniques, and a machine learning algorithm (a support vector machine classifier). While this proposal is related to ours, we also focus on building a corpus from ProMED-mail articles on diseases prevalent in Latin America and in Spanish. Besides, we share this corpus -as far as we know, the first available corpus in Spanish for this goal- with the purpose of providing more opportunities to do open science.

## Conclusion

Event-based systems provide information that may account for important events of public health, often through channels outside of routine surveillance, such as social media. However, while there are several social media sources for disseminating information about disease surveillance, there is a lack of tools that can automatically provide detailed epidemiological data. This is often most noticeable when considering media sources produced in the Spanish language.

In this context, the aim of this paper was to provide tools that enable disease surveillance of outbreak diseases prevalent in Latin America from text written in Spanish. Thus, we created an annotated corpus based on ProMED-mail articles, that is useful for automatically extracting epidemiological data from newspaper articles written in Spanish. We have also developed two named entity recognizers -one of them based on Flair (a neural network) and the other based on rules-, and a baseline algorithm for relation extraction based on the co-occurrence of named entities. As expected, the neural network approach for NER has better results than the rule-based method. On the other hand, it is an expensive method, due to the need of having annotated data. The annotation process was expensive in terms of resources: an annotation criteria had to be created and annotators had to be found and trained. Moreover, the fact of not being able to economically compensate annotators made that some of them could not remain throughout the whole project, which extended the annotation time and lowered the quality of the created corpus. Besides, the fact of working with annotators of different research areas (computer scientists and linguists) made it more interesting but also much more challenging, because of the different backgrounds. Furthermore, the existence of some annotation errors interfered with the results of the algorithms. Nevertheless, it is important to notice that inter-annotator agreement measures are better than those shown since in our implementation we only count for those annotations that have exact coincidences (partial coincidences were not taken into account).

For creating the corpus we analyzed the convenience of working with only articles’ titles and dates (Titles) or complete articles (PMAs). We concluded that in order to extract information of dates, diseases, locations, and where a disease was located it would suffice with analyzing Titles. This is an important finding since it is much easier to only annotate Titles than complete articles. The advantage of working with complete articles instead of only titles and dates is that more entities and relations can be found in PMAs, which allows for a more detailed analysis of outbreaks.

We also analyzed two ways of evaluation of our NER algorithms: the traditional exact match and a partial match, which allows to count matches that are not total, but that are relatively good guesses. In our opinion, the second metric is more appropriate for this problem.

Our study has some limitations. First of all, since -as far as we know- there are no publicly available corpora, and no other work for Spanish, we can not compare our results to others. Besides, the size of our corpus (513 articles) constitutes a limitation for the application of other deep learning methods.

For future work, one possible and easy improvement would be to improve annotations by eliminating those of one of the annotators, that did not follow the annotation criteria. Also, the extracted information could be used to provide a map with worldwide information on the appearance of different diseases, like those provided by Johns Hopkins University.^24^

Overall, we consider that our systems and resources are the first steps to arrive at a fully automated system and contribute to the purpose of doing open science, given that they might help other researchers to make progress in this research area. In particular, other researchers could benefit of (1) the corpus, as far as we know, the first available corpus in Spanish, for the development of their own algorithms and for comparing the results of new algorithms with ours, (2) the annotation criteria, described by us in another paper ([10]), in order to generate their own corpus, and (3) the Spanish NER and RE algorithms provided, that have good results, and for which, as far as we know, there were no previous works, in order to use them as baselines or to compare them with other methods.

## Data Availability

The datasets generated and analysed during the current study are available at https://sites.google.com/view/spanishcorpusoutbreakdetection/home.

## Footnotes

1 An early version of a guide of PAHO and WHO to the implementation of surveillance capacity, especially EBS, in order to rapidly detect and respond to all acute health events and risks of any origin, written in Spanish is available at https://iris.paho.org/handle/10665.2/10115?locale-attribute=en. Last accessed: August 2022.

2 IHR: https://www.who.int/publications/i/item/9789241580496. Last accessed: August 2022.

3 CDC Zika health effects: https://www.cdc.gov/zika/healtheffects/gbs-qa.html#:~:text=Guillain%2DBarr%C3%A9%20syndrome%20(GBS),weakness%2C%20and%20sometimes%2C%20paralysis.. Last accessed: August 2022.

4 Particularly, one of the missions of PAHO is to promote the development of surveillance systems and epidemiology analysis in order to reduce the prevalence caused by communicable diseases in the Region. Pan American Health Organization. Neglected, Tropical and Vector-Borne Diseases. https://www.paho.org/en/topics/neglected-tropical-and-vector-borne-diseases. Last accessed: August 2022.

5 Epidemiological Update: Measles. PAHO. November 2021. https://iris.paho.org/handle/10665.2/55325. Last accessed: August 2022.

6 The corpus can be accessed through the following webpage: https://sites.google.com/view/spanishcorpusoutbreakdetection/home.

7 The corpus presented in the mentioned paper was enhanced in this paper.

8 Eg. REC, epidemiological report from Córdoba, Argentina: http://www.reporteepidemiologico.com.ar/ and from the whole country https://bancos.salud.gob.ar/bancos/materiales-para-equipos-de-salud/soporte/boletines-epidemiologicos/.

9 MedISys: https://medisys.newsbrief.eu/medisys/homeedition/en/home.html.

10 PULS: http://puls.cs.helsinki.fi/static/index.html.

11 SENTINEL SURVEILLANCE: https://www.ecdc.europa.eu/en/seasonal-influenza/surveillance-and-disease-data/facts-sentinel-surveillance.

12 GRITS: https://www.ecohealthalliance.org/program/grits.

13 ProMED-mail: https://promedmail.org/about-promed/.

14 scikit-learn https://scikit-learn.org/stable/.

15 The thesis is written in Spanish.

16 GeoNames WebServices. Available at: http://www.geonames.org/export[Accessed Octobe 2020].

17 This decision was taken based on the Gazetters’ information.

18 Trained with different algorithms: fastText, Glove and Word2Vec.

19 Using the forward and backward embeddings, in its normal and *pooled* version.

20 The average length of a word of our corpus is of five characters.

21 Dashes (-) indicate that the algorithm does not detect that type of entity. Zeroes indicate that the entity is detected, but wrongly in all cases.

22 Some terms are annotated as two different entities (eg. Zika as “origin” and as “disease”). In those cases, when transforming a .ann file into a conll format, one of the labels is lost, due that conll does not accept multiple labels. In this particular case, one of the entities was labeled as disease, and origin was discarded. For that reason, recall is 0.2.

23 In titles, the RNN method does not detect entities of type “Negation” and “Past”.

24 https://coronavirus.jhu.edu/map.html.

## References

[1] Balajee SA, Salyer SJ, Greene-Cramer B, Sadek M, Mounts AW (2021). The practice of event-based surveillance: concept and methods. Global Secur Health Sci Policy.

[2] O’Shea J (2017). Digital disease detection: a systematic review of event-based internet biosurveillance systems. Int J Med Inform.

[3] Ganser I. Evaluation of event-based internet biosurveillance for multi-regional detection of seasonal influenza onset. Master’s thesis, The Digital Public Health Graduate Program, University of Bordeaux. 2020.

[4] Zeng D, Cao Z, Neill DB (2021). Artificial intelligence-enabled public health surveillance-from local detection to global epidemic monitoring and control. Artif Intell Med.

[5] Gupta A, Katarya R (2020). Social media based surveillance systems for healthcare using machine learning: a systematic review. J Biomed Inform.

[6] Freifeld CC, Mandl KD, Reis BY, Brownstein JS (2008). Healthmap: global infectious disease monitoring through automated classification and visualization of internet media reports. J Am Med Inform Assoc.

[7] Ng V, Rees EE, Niu J, Zaghool A, Ghiasbeglou H, Verster A (2020). Application of natural language processing algorithms for extracting information from news articles in event-based surveillance. Can Commun Dis Rep.

[8] Baclic O, Tunis M, Young K, Doan C, Swerdfeger H, Schonfeld J (2020). Artificial intelligence in public health: challenges and opportunities for public health made possible by advances in natural language processing. Can Commun Dis Rep.

[9] Carrion M, Madoff LC (2017). ProMED-mail: 22 years of digital surveillance of emerging infectious diseases. Int Health.

[10] Dellanzo A, Cotik V, Ochoa-Luna J. A corpus for outbreak detection of diseases prevalent in Latin America. In: Proceedings of the 24th conference on computational natural language learning . Association for Computational Linguistics; 2020. pp 543–51. 10.18653/v1/2020.conll-1.44. https://aclanthology.org/2020.conll-1.44

[11] Charles-Smith LE, Reynolds TL, Cameron MA, Conway M, Lau EH, Olsen JM, Pavlin JA, Shigematsu M, Streichert LC, Suda KJ, Corley CD (2015). Using social media for actionable disease surveillance and outbreak management: a systematic literature review. PLoS ONE.

[12] Thiebaut R, Cossin S (2019). Artificial intelligence for surveillance in public health. Yearb Med Inform.

[13] Edo-Osagie O, De La Iglesia B, Lake I, Edeghere O (2020). A scoping review of the use of Twitter for public health research. Comput Biol Med.

[14] Rolland C, Lazarus C, Giese C, Monate B, Travert AS, Salomon J (2020). Early detection of public health emergencies of international concern through undiagnosed disease reports in ProMED-mail. Emerg Infect Dis.

[15] Bonilla-Aldana DK, Holguin-Rivera Y, Cortes-Bonilla I, Cardona-Trujillo MC, García-Barco A, Bedoya-Arias HA, Rabaan AA, Sah R, Rodriguez-Morales AJ (2020). Coronavirus infections reported by ProMED. Travel Med Infect Dis.

[16] Simmie D, Thapen N, Hankin C. DEFENDER: detecting and forecasting epidemics using novel data-analytics for enhanced response. 2015. arXiv preprint arXiv:1504.04357. 10.1371/journal.pone.0155417 PMC4871418 27192059

[17] Abbood A, Ullrich A, Busche R, Ghozzi S (2020). EventEpi-A natural language processing framework for event-based surveillance. PLoS Comput Biol.

[18] Arsevska E, Valentin S, Rabatel J, de Hervé JG, Falala S, Lancelot R, Roche M (2018). Web monitoring of emerging animal infectious diseases integrated in the French animal health epidemic intelligence system. PLoS ONE.

[19] Cotik V, Alemany LA, Filippo D, Luque F, Roller R, Vivaldi J, Ayach A, Carranza F, Francesca L, Dellanzo A et al. Overview of CLEF eHealth Task 1-SpRadIE: a challenge on information extraction from Spanish radiology reports. In: CLEF 2021 evaluation labs and workshop: online working notes. CEUR-WS; 2021.

[20] Piad-Morffis A, Estevez-Velarde S, Gutierrez Y, Almeida-Cruz Y, Montoyo A, Muñoz R (2021). Overview of the ehealth knowledge discovery challenge at iberlef 2021. Procesamiento del Lenguaje Natural.

[21] Bender EM, Friedman B (2018). Data statements for natural language processing: toward mitigating system bias and enabling better science. Trans Assoc Comput Linguist.

[22] Ide N, Pustejovsky J. Handbook of linguistic annotation. Springer; 2017.

[23] Stenetorp P, Pyysalo S, Topić G, Ohta T, Ananiadou S, Tsujii J. brat: a web-based tool for NLP-assisted text annotation. In: Proceedings of the demonstrations session at EACL 2012. Association for Computational Linguistics, Avignon, France; 2012.

[24] Cohen J (1960). A coefficient of agreement for nominal scales. Educ Psychol Measur.

[25] Dellanzo A. Detección de epidemias en textos periodísticos escritos en español. In: Master thesis. Universidad de Buenos Aires; 2021.

[26] Padró L, Stanilovsky E. FreeLing 3.0: towards wider multilinguality. In: Proceedings of the language resources and evaluation conference (LREC 2012). ELRA, Istanbul, Turkey; 2012.

[27] Akbik A, Blythe D, Vollgraf R. Contextual string embeddings for sequence labeling. In: COLING 2018, 27th international conference on computational linguistics. 2018. p. 1638–49.

[28] Reimers N, Gurevych I. Optimal hyperparameters for deep LSTM-networks for sequence labeling tasks. 2017. arXiv preprint arXiv:1707.06799.

[29] Kingma DP, Ba J. Adam: a method for stochastic optimization. 2014. arXiv preprint arXiv:1412.6980.

[30] Cardellino C. Spanish Billion Words Corpus and Embeddings; 2019. https://crscardellino.github.io/SBWCE/.

[31] Cotik V. Information extraction from Spanish radiology reports. In: Ph.D thesis. Universidad de Buenos Aires; 2018.

[32] Chinchor N, Lewis DD, Hirschman L. Evaluating message understanding systems: an analysis of the third message understanding conference (MUC-3). Science Applications International Corp San Diego, CA: Technical report; 1993.

